# Pathology re-review as an essential component of breast cancer management

**DOI:** 10.3747/co.v17i1.517

**Published:** 2010-02

**Authors:** C.G. Kleer

Approximately one third of cancers in women arise in the breast, making breast cancer the most commonly diagnosed cancer by far 1. Not only does breast cancer affect many women, it is also a concern for all women. This situation emphasizes the importance of knowledge about breast cancer for all health care professionals who are responsible for the care of women. This central concept drives the multidisciplinary approach to breast cancer management. In the multidisciplinary model, pathologists are an integral and essential component of a team of health care professionals who work together to provide the best treatment available for every patient with breast cancer.

In this issue of *Current Oncology,* the study by Price *et al.* 2 underscores the role of pathology re-review in treatment decisions for breast cancer patients. That study was conducted at the QEII Health Sciences Centre in Halifax, Nova Scotia, which is a tertiary oncology referral centre. Thus, after initial diagnosis or surgery, breast cancer patients are referred to QEII for adjuvant medical and radiation oncology evaluation. In the Price *et al.* study of 93 cases, 10 cases (11%) underwent a change in diagnosis deemed to have medium or high impact for differences in clinical management. This rate of discrepancy accords with rates identified in previous studies, which range from 1.4% to 29% 3,4.

Which were the main discrepancies observed? Not surprisingly, no unique cause prompted a change in diagnosis. The most significant discrepancies between the first diagnosis and the second pathology review at QEII—those classified as having a high clinical impact—were changes in diagnosis from ductal carcinoma *in situ* to invasive carcinoma; differences in measured tumour size, placing the tumour in a different staging category; differing interpretations of hormone receptor status; and differing interpretations of isolated tumour cells, resulting in changed nodal status. Discrepancies classified as having medium clinical impact primarily involved margin status assessment.

The Price *et al.* study, together with previous studies on the topic, highlights the importance of pathology re-review before treatment recommendations are made in breast cancer and shows how re-review is likely applicable to other malignancies.

Can we avoid disagreements in pathology interpretations? Despite adequate pathology training and experience, disagreements in diagnosis are unavoidable. This situation stems from the difficulties inherent to practice, as well as from different abilities and personal interpretations regarding tissue changes. It should also be kept in mind that subtle changes in pathologic interpretation may have profound clinical implications.

A particular challenge in breast pathology is the importance of taking multiple accurate measurements (tumour, margins, metastasis), which most of the time are not straightforward and are crucial for treatment purposes. Another challenge for breast pathologists is the interpretation of estrogen and progesterone receptor status and analysis of human epidermal growth factor receptor (her2/*neu*) amplification and overexpression. Despite several published guidelines, these tests are performed and interpreted in different ways by different laboratories.

Given the foregoing considerations, I believe that we can improve our practice in several ways, which are also highlighted by the data provided by Price and coworkers. One of the most important ways in which breast pathology interpretation can be made more accurate is through the use of synoptic diagnostic reports and templates. These tools guide the pathologist concerning the information that is necessary in the report and use a more standard language that more effectively communicates to oncologists, radiologists, and surgeons. Another way in which breast pathology practice can be improved is adherence to published guidelines on the interpretation of hormone receptor and her2/*neu* status 5,6.

Of critical importance in the current management of breast cancer patients is effective communication between the pathologist and the rest of the multidisciplinary team, including primary care physicians (internists, family medicine specialists, nurses), geneticists, breast radiologists, breast pathologists, surgical breast specialists, and radiation and medical oncology specialists. Tumour boards provide a forum for discussing cases, with the aim of planning treatment. Central pathology review of each case by an expert pathologist before the multidisciplinary meeting provides the opportunity to re-evaluate diagnoses and to identify any discrepancies that may influence management. The results from the study by Price and colleagues are in agreement with previous literature on the subject 3,4 and, together with those other studies, demonstrate that the multimodality approach with pathology re-review can provide additional useful information for therapeutic decision-making.
